# Oral Food-Derived Proanthocyanidins as Immunostimulants: Enhanced Vaccine Immunogenicity via Host Immune Modulation in a Murine CSFV Model

**DOI:** 10.3390/molecules31162766

**Published:** 2026-08-09

**Authors:** Ke Yue, Yanzhi Zhang, Xing Zhang, Kunmiao He, Chong Yuan, Jiusi Chen, Hongtao Ren, Na Wang, Gaiping Zhang

**Affiliations:** 1 Longhu Laboratory, Zhengzhou 450046, China; yueke202201@163.com (K.Y.); aa764203299@163.com (Y.Z.); a1009620039@gmail.com (X.Z.); 18338397558@163.com (K.H.); yc17637476643@163.com (C.Y.); zzjschen2022@163.com (J.C.); zhanggaiping@pku.edu.cn (G.Z.); 2 International Joint Research Center of National Animal Immunology, College of Veterinary Medicine, Henan Agricultural University, Zhengzhou 450046, China; 3 Zhengzhou Key Laboratory of Nutrition and Health Food, College of Food Science and Technology, Henan Agricultural University, Zhengzhou 450002, China; 4 School of Advanced Agriculture Sciences, Peking University, Beijing 100871, China

**Keywords:** proanthocyanidins (PACs), oral immunostimulants, host immune conditioning, Th1/Th2 balanced immunity, food-derived natural products

## Abstract

Traditional injectable vaccine adjuvants are limited by local granulomatous reactions, chronic inflammation, and reduced compliance, creating an urgent need for safe, host-directed strategies to enhance vaccine immunogenicity. Proanthocyanidins (PACs)—natural polyphenols abundant in grapes, cocoa, and tea—are food-derived bioactive compounds with antioxidant, anti-inflammatory, and immunomodulatory activities, positioning them as attractive candidates for oral immunostimulants. Using classical swine fever virus (CSFV), a Pestivirus within the Flaviviridae that shares conserved genomic and immunological features with human pathogens such as hepatitis C and dengue virus, we systematically evaluated oral PACs (15, 30, and 60 mg/kg/d) combined with attenuated live or subunit CSFV vaccines in mice. PACs were well tolerated across the full dose range, with no impact on body weight or major organs, while selectively increasing the splenic index—suggesting spleen-targeted immune activation without systemic inflammation. Compared with vaccine alone, the moderate dose (30 mg/kg/d) produced the strongest immunostimulation, particularly with the subunit vaccine: peak antigen-specific IgG titer reached 1:409,600, a 4-fold increase versus 1:102,400 for subunit alone and exceeding the 3.4-fold gain observed with the attenuated vaccine. IgG2a and IgG2b rose by 199.4% and 269.1%, respectively, alongside coordinated elevations of Th1 (IL-2 +160.6%, IFN-γ +91.9%) and Th2 (IL-4, IL-10) cytokines, enhanced B- and T-lymphocyte proliferation, and expansion of CD4^+^ and CD8^+^ subsets, establishing a balanced and durable Th1/Th2 response. These findings provide preclinical evidence that food-derived PACs can serve as safe oral immunostimulants for low-immunogenicity subunit vaccines, supporting their further development in human and veterinary vaccinology.

## 1. Introduction

Dietary proanthocyanidins (PACs) are a class of polyphenolic compounds widely found in natural foods such as grapes and cocoa beans. They form oligomers or polymer chains through specific covalent bonds between flavan-3-ol monomers and are among the most common phenolic compounds in the human diet [1,2]. In addition to their established antioxidant activity, their significant immunostimulatory properties make them ideal natural candidates for the development of oral immune-enhancing strategies [3,4,5]. Unlike traditional injectable vaccine adjuvants, orally administered PACs may provide a safe and convenient complementary approach for supporting vaccine-induced immune responses, although the biological processes underlying this association remain to be determined.

Although vaccines are the most effective means of preventing infectious diseases, there are significant differences in immunogenicity among different types of vaccines, a challenge that is particularly pronounced in the development of classical swine fever virus (CSFV) vaccines. Although live-attenuated CSFV vaccines can induce strong immunoprotection, they carry a risk of virulence resurgence; conversely, while CSFV subunit vaccines offer superior safety, their relatively weak immunogenicity often requires multiple doses supplemented with effective adjuvants [6,7]. However, the safety profiles of traditional adjuvants remain inadequate: aluminum-based adjuvants often induce granuloma formation at the injection site, while oil-in-water emulsions may trigger excessive inflammation [8,9]. Such adverse reactions not only compromise the safety of vaccination but also exacerbate vaccine hesitancy worldwide. Therefore, exploring safe strategies that holistically enhance the immune response at the host level has become a critical research direction for optimizing vaccine efficacy.

CSFV belongs to the genus Pestivirus within the family Flaviviridae. Its molecular biological characteristics share several conserved features with those of important human pathogens such as hepatitis C virus and dengue virus, including genome organization, protein processing, and replication mechanisms. Furthermore, these viruses have evolved complex immune evasion strategies, presenting common technical challenges for vaccine development [10,11]. Effective immune protection against CSFV and human RNA viruses requires the balanced activation of humoral and cellular immunity, particularly the synergistic action of CD4^+^ and CD8^+^ T cells [12]. This requires the host’s immune system to be in an optimal state of responsiveness at the time of vaccination in order to achieve an ideal balance between Th1 and Th2 responses—and this is precisely where PACs can play a unique role as immunomodulators.

Food-derived PACs, as natural oral immunomodulators, offer unique advantages in optimizing the immunogenicity of vaccination at the host level. Their immunomodulatory mechanisms are highly conserved in both humans and animals; following oral administration, PACs and/or their gut-microbiota-derived metabolites may modulate systemic immune function, potentially influencing dendritic cell activity, cytokine secretion, and T-cell differentiation [13,14]. These previously reported immunomodulatory properties raise the possibility that oral PAC administration may influence the magnitude or profile of vaccine-induced immune responses. More importantly, with a safety record validated by thousands of years of human consumption, the oral route of administration also avoids the local adverse reactions commonly associated with injectable adjuvants, making it a safe, convenient, and cross-species-transferable immunostimulatory strategy [15].

Based on the above analysis, this study uses CSFV as a translational medicine model and, following the “nutritional immunology” research paradigm, focuses on investigating the immunostimulatory effects of oral PACs on the immune response to CSFV vaccines. We systematically evaluated whether oral PAC administration was associated with changes in vaccine-induced humoral and cellular immune readouts in mice receiving either an attenuated live or a subunit CSFV vaccine. The assessed outcomes included antigen-specific IgG and IgG subclasses, serum cytokines, splenic B- and T-lymphocyte proliferation, and peripheral CD4^+^ and CD8^+^ T-cell proportions. These findings provide preclinical evidence supporting further investigation of food-derived PACs as candidate oral immunostimulants in vaccination settings.

## 2. Results

### 2.1. Effects of Pacs on Baseline Physiological Parameters and Antibody Responses in Mice Immunized with Attenuated Csfv Vaccine

**Figure 1:**
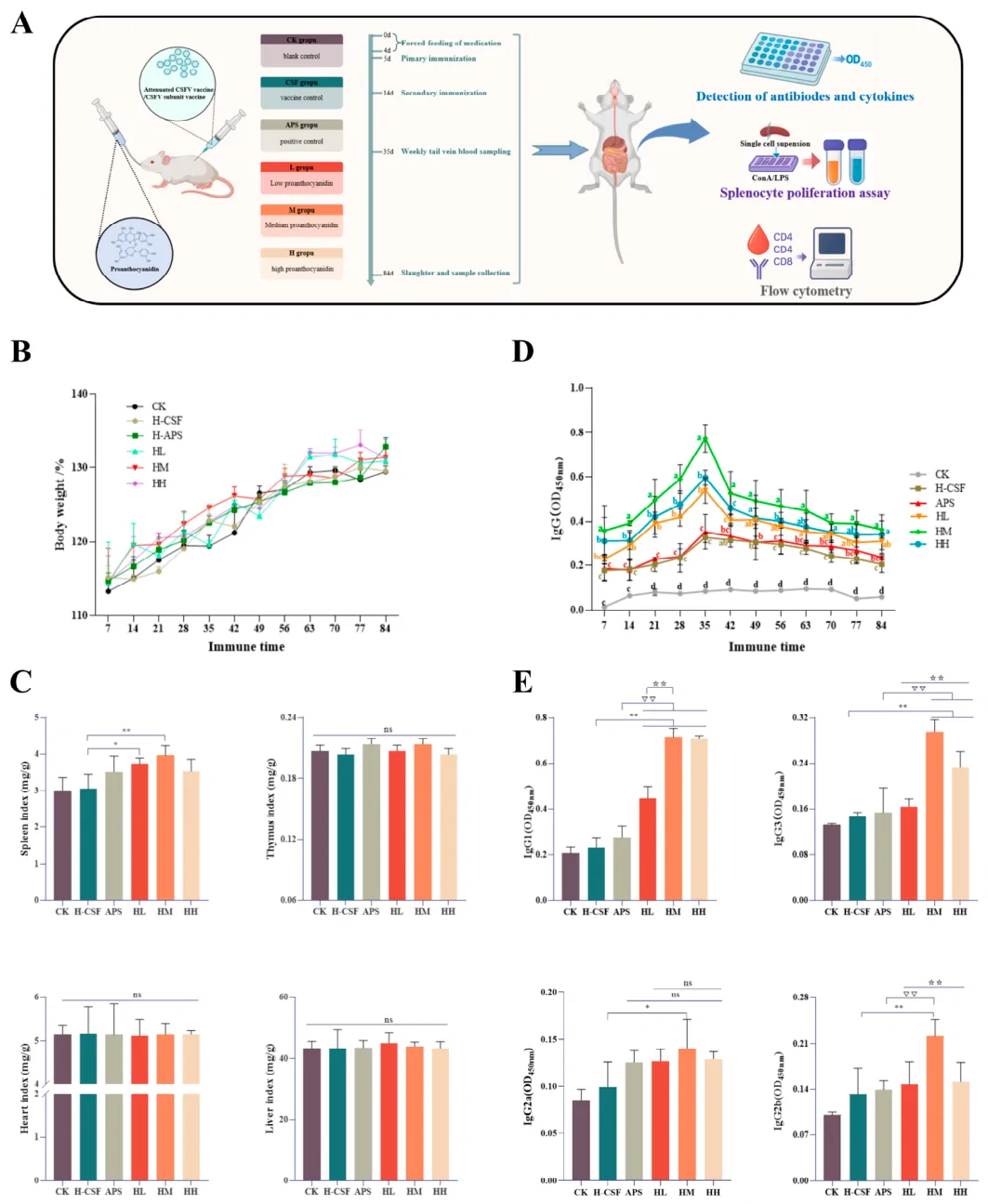
Effects of PACs on baseline physiological parameters and antibody responses in mice immunized with attenuated CSFV vaccine. (**A**) Schematic Diagram of the Experimental Procedure. (**B**) Changes in body weight of mice in different treatment groups at various time points post-immunization. (**C**) Organ indices of spleen, thymus, heart, and liver in mice. (**D**) Dynamic changes in serum IgG levels of mice at different time points post-immunization. Different lowercase letters indicate significant differences among groups at the same time point (*p* < 0.05), whereas groups sharing the same letter are not significantly different. (**E**) Serum IgG subclasses (IgG1, IgG3, IgG2a, and IgG2b) on day 35 after booster immunization. Note: compared with the H-CSF group, * *p* < 0.05, ** *p* < 0.01; compared with the APS group, ^▽^ *p* < 0.05, ^▽▽^ *p* < 0.01; compared with the HM group, ^☆^ *p* < 0.05, ^☆☆^ *p* < 0.01.

The body weight of mice administered different doses of PACs showed no significant fluctuations throughout the experimental period, and there was no significant difference compared with the CK group, indicating that PACs did not adversely affect the normal growth and development of mice and exhibited good biosafety (Figure 1A). Further analysis of organ indices (Figure 1B) revealed that the most pronounced effect of PACs was observed in the spleen. Specifically, compared with the H-CSF group, the splenic index of mice in the low-dose group (15 mg/kg/d) was significantly increased (*p* < 0.05), while the medium-dose group (30 mg/kg/d) showed an even greater increase with highly significant differences (*p* < 0.01). The high-dose group (60 mg/kg/d) exhibited an increasing trend, although the difference was not statistically significant. Compared with the APS group, no significant differences were observed among the three proanthocyanidin-treated groups (*p* > 0.05). Moreover, no significant differences were detected among the three dose groups of PACs themselves (*p* > 0.05). In contrast, PACs at different doses had no significant effects on the indices of the thymus, heart, or liver (*p* > 0.05), indicating that PAC administration was associated with an increased spleen index in selected groups, whereas the indices of the other examined organs remained unchanged.

Changes in specific IgG levels at different time points after the booster immunization are shown in Figure 1C. On day 7, serum specific IgG levels in the medium- and high-dose proanthocyanidin groups were significantly higher than those in the H-CSF group (*p* < 0.05). The APS group and the low-dose group also showed an upward trend, although the differences were not significant (*p* > 0.05). Compared with the APS group, IgG levels in the medium- and high-dose groups were also significantly elevated (*p* < 0.05). From day 14 to day 35, IgG levels in all groups continued to rise and reached a peak on day 35. During this period, the low-, medium-, and high-dose proanthocyanidin groups all showed significantly higher IgG expression compared with both the vaccine and APS groups (*p* < 0.05), indicating a sustained immunoenhancing effect. From day 42 onward, IgG levels began to decline; however, between days 70 and 84, IgG levels in all three proanthocyanidin groups remained significantly higher than those in the H-CSF group (*p* < 0.05), with the medium- and high-dose groups also exceeding the APS group (*p* < 0.05). Notably, no significant differences were observed among the three proanthocyanidin dose groups during this maintenance phase (*p* > 0.05), suggesting that dose-dependent differences diminished at later time points.

Further analysis of IgG subclasses is shown in Figure 1D. For IgG1, the low-, medium-, and high-dose proanthocyanidin groups were all significantly higher than the H-CSF group (*p* < 0.01), whereas the APS group showed an increase without reaching significance (*p* > 0.05). For IgG3, the medium- and high-dose groups exhibited highly significant increases compared with both the vaccine and APS groups (*p* < 0.01), while the low-dose group showed a non-significant upward trend. For IgG2a, only the medium-dose group showed a significant increase compared with the H-CSF group (*p* < 0.05), with no significant differences among the other groups (*p* > 0.05). For IgG2b, the medium-dose group was highly and significantly elevated compared with both the vaccine and APS groups (*p* < 0.01), whereas the low- and high-dose groups showed increases that were not statistically significant. Collectively, these findings indicate that the IgG subclass results further support the superior effect of medium-dose PACs in enhancing humoral immunity, particularly by promoting IgG1, IgG3, and IgG2b expression.

### 2.2. Effects of Pacs on Immune Responses in Mice Immunized with Attenuated Csfv Vaccine

**Figure 2:**
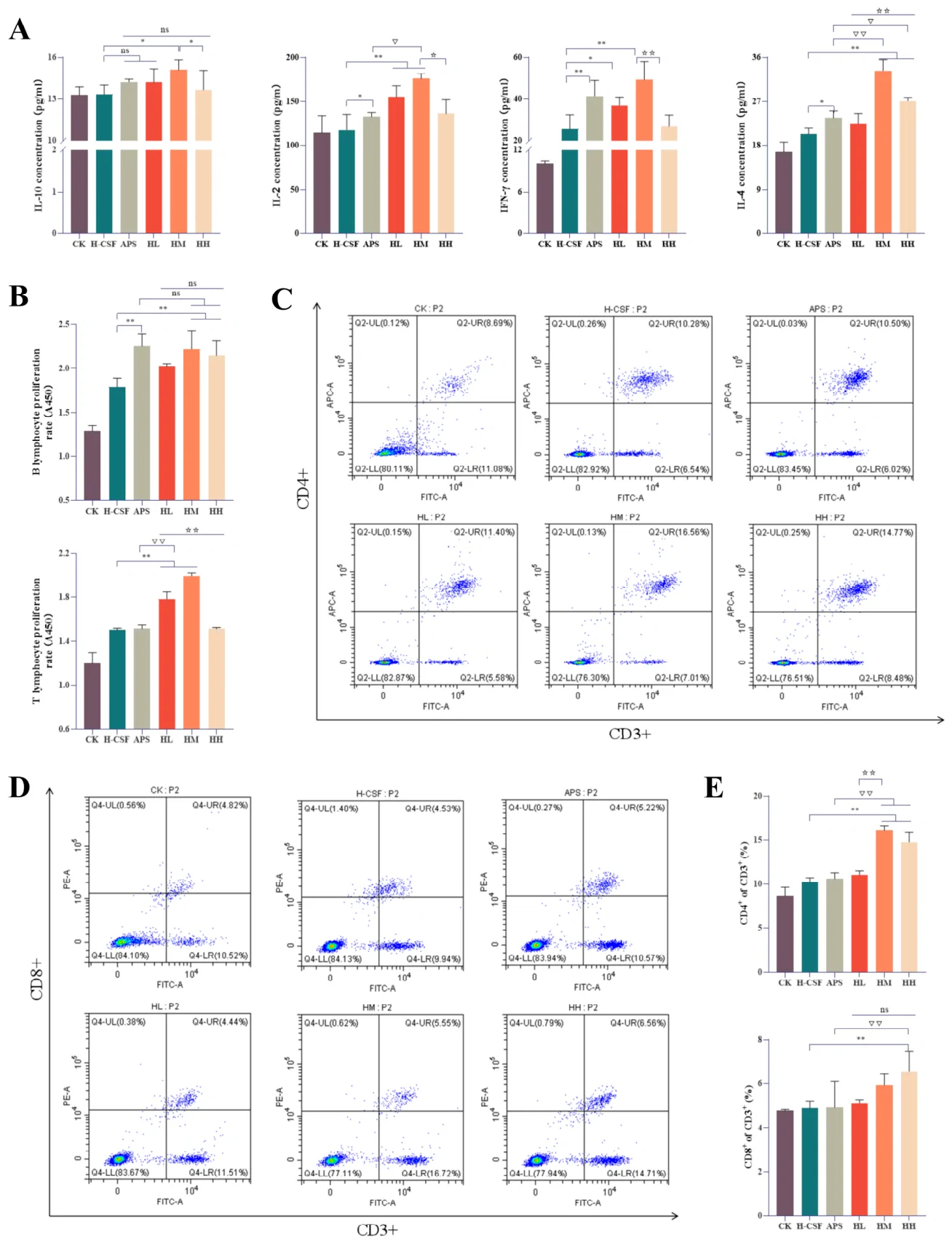
Effects of PACs on immune responses in mice immunized with attenuated CSFV vaccine. (**A**) Serum concentrations of IL-10, IL-2, IFN-γ, and IL-4 on day 35 after booster immunization. (**B**) Proliferation rates of splenic B lymphocytes and T lymphocytes on day 35 after booster immunization. (**C**,**D**) Flow cytometric analysis of CD4^+^ (**C**) and CD8^+^ (**D**) T-cell subsets in peripheral blood. In the pseudocolor plots, cooler and warmer colors indicate lower and higher event densities, respectively. (**E**) Ratios of CD4^+^/CD3^+^ and CD8^+^/CD3^+^ T cells in peripheral blood on day 35 after booster immunization. Note: compared with the H-CSF group, * *p* < 0.05, ** *p* < 0.01; compared with the APS group, ^▽^ *p* < 0.05, ^▽▽^ *p* < 0.01; compared with the HM group, ^☆^ *p* < 0.05, ^☆☆^ *p* < 0.01.

To evaluate the immunomodulatory effects of PACs combined with the attenuated CSFV vaccine, serum levels of four representative cytokines (IL-4, IL-10, IL-2, and IFN-γ) were measured in mice (Figure 2A). IL-4 and IL-10 are typical Th2-type cytokines that enhance humoral immune responses and promote antibody production, whereas IL-2 and IFN-γ primarily reflect Th1-type immunity and are closely associated with T-cell activation and cytotoxic responses. The results showed that, compared with the H-CSF group, the APS group exhibited significantly elevated IL-4 levels (*p* < 0.05), while both the medium- and high-dose proanthocyanidin groups displayed highly significant increases (*p* < 0.01). Among them, the medium-dose group (30 mg/kg/d) was significantly higher than the high-dose group (*p* < 0.01) and also significantly higher than the APS group (*p* < 0.01), indicating the strongest effect on humoral immune regulation. For IL-10, only the medium-dose group showed a significant increase compared with the H-CSF group (*p* < 0.05), whereas no significant differences were observed among the other groups (*p* > 0.05). Although proanthocyanidin-treated groups exhibited upward trends compared with the APS group, the differences were not significant (*p* > 0.05). Notably, IL-10 levels in the medium-dose group were significantly higher than those in the high-dose group (*p* < 0.05). Regarding IFN-γ, both the APS group and the medium-dose proanthocyanidin group showed highly significant increases compared with the H-CSF group (*p* < 0.01), while the low-dose group also showed a significant increase (*p* < 0.05). Furthermore, IFN-γ levels in the medium-dose group were significantly higher than those in the high-dose group (*p* < 0.01), suggesting the strongest activation of the Th1 immune response in the medium-dose group. For IL-2, significant increases were observed in the APS group (*p* < 0.05) and in the medium- and high-dose groups (*p* < 0.01). IL-2 levels in the medium-dose group were significantly higher than those in the high-dose group (*p* < 0.05). Taken together, the medium-dose proanthocyanidin group demonstrated the most pronounced immunoenhancing effect across all four cytokine indicators (IL-4, IL-10, IFN-γ, and IL-2), with particularly strong activity in promoting Th2-mediated immune responses.

The regulatory effects of PACs on lymphocyte function were further evaluated using splenic lymphocyte proliferation assays (Figure 2B). For B lymphocytes, compared with the H-CSF group, both the APS group and the medium- and high-dose proanthocyanidin groups showed highly significant increases in proliferation rates (*p* < 0.01). No significant differences were observed between the proanthocyanidin-treated groups and the APS group (*p* > 0.05), nor among the three dose groups themselves (*p* > 0.05). For T lymphocytes, the low- and medium-dose proanthocyanidin groups exhibited highly significant increases compared with both the vaccine and APS groups (*p* < 0.01). Notably, the proliferation rate in the medium-dose group was significantly higher than those in the low- and high-dose groups (*p* < 0.01), suggesting a dose-related promoting effect.

To further analyze changes in T-cell subsets, flow cytometry was used to determine the proportions of CD3^+^CD4^+^ and CD3^+^CD8^+^ T lymphocytes (Figure 2E). For CD3^+^CD4^+^ T cells, both the medium- and high-dose proanthocyanidin groups exhibited highly significant increases compared with the H-CSF group (*p* < 0.01), and also showed highly significant differences relative to the APS group (*p* < 0.01). Moreover, the proportion of CD4^+^ T cells in the medium-dose group was significantly higher than those in the low- and high-dose groups (*p* < 0.01). These results indicate that medium-dose PACs possess strong helper T-cell activating capacity, which may mobilize downstream cytokine networks and enhance humoral immune responses. For CD3^+^CD8^+^ T cells, only the high-dose proanthocyanidin group showed a highly significant increase compared with both the vaccine and APS groups (*p* < 0.01). Although other groups exhibited upward trends, the differences were not statistically significant (*p* > 0.05), suggesting that higher doses may preferentially promote cytotoxic T-cell activation. This pattern is consistent with the earlier observation that the medium dose favors Th2 responses, whereas the high dose tends to enhance CD8^+^ T-cell activation.

### 2.3. Effects of Pacs on Baseline Physiological Parameters and Antibody Responses in Mice Immunized with a Subunit Vaccine Against Csfv

**Figure 3:**
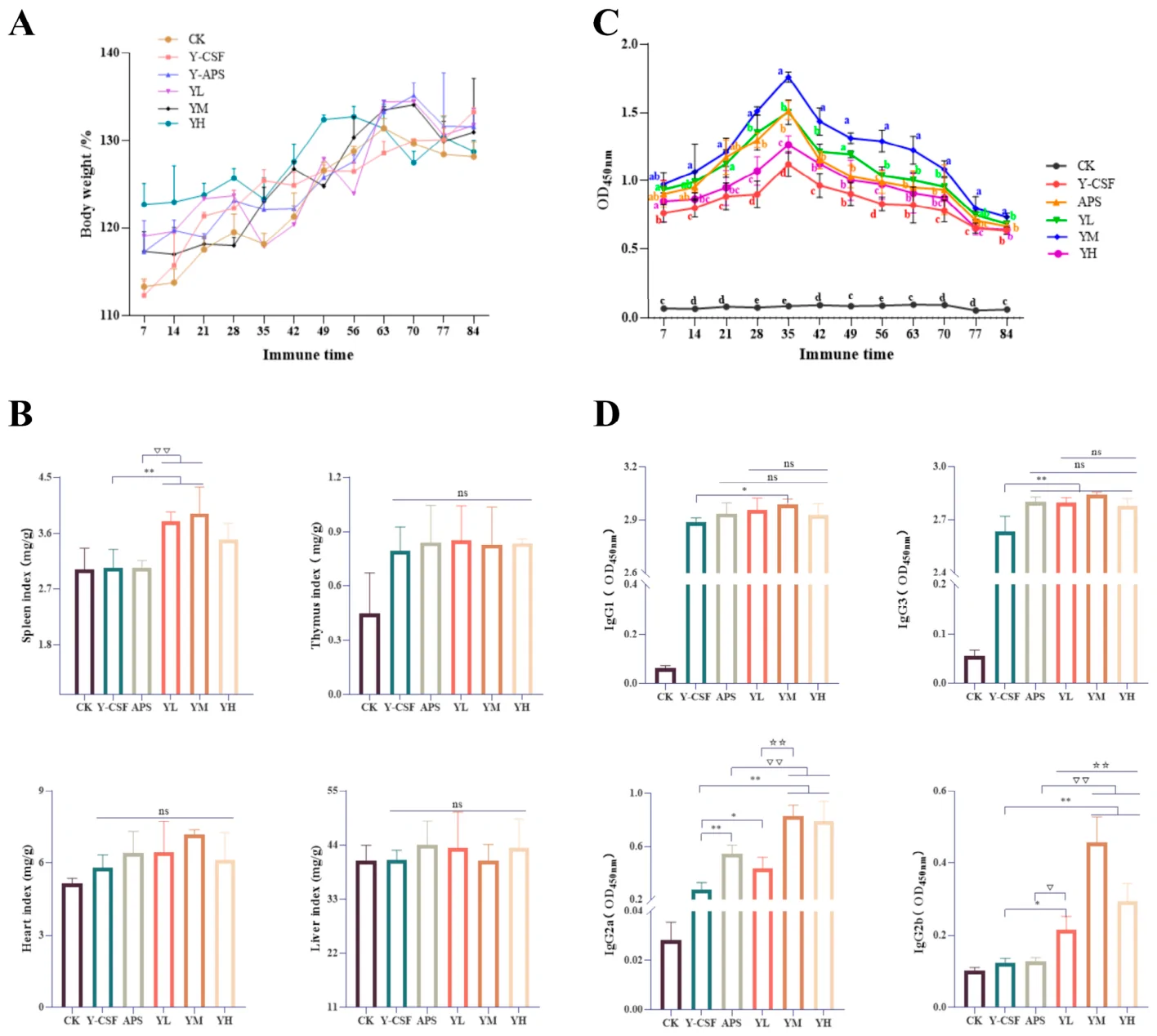
Effects of PACs on baseline physiological parameters and antibody responses in mice immunized with a subunit vaccine against CSFV. (**A**) Changes in body weight of mice in different treatment groups at various time points post-immunization. (**B**) Organ indices of spleen, thymus, heart, and liver in mice. (**C**) Dynamic changes in serum IgG levels of mice at different time points post-immunization. Different lowercase letters indicate significant differences among groups at the same time point (*p* < 0.05), whereas groups sharing the same letter are not significantly different. (**D**) Serum IgG subclasses (IgG1, IgG3, IgG2a, and IgG2b) on day 35 after booster immunization. Note: compared with the Y-CSF group, * *p* < 0.05, ** *p* < 0.01; compared with the APS group, ^▽^ *p* < 0.05, ^▽▽^ *p* < 0.01; compared with the YM group, ^☆^ *p* < 0.05, ^☆☆^ *p* < 0.01.

As shown in Figure 3A, oral administration of different doses of PACs had no significant effect on body weight. No significant differences were observed between any treatment group and the CK group (*p* > 0.05), indicating that PACs did not impair normal growth and development and exhibited good biosafety. Further analysis of organ indices (Figure 3B) revealed that the splenic index of mice in the low- and medium-dose groups was significantly increased compared with the Y-CSF group (*p* < 0.01). Moreover, both the medium- and high-dose groups showed highly significant increases compared with the APS group (*p* < 0.01), suggesting a marked regulatory role in splenic development or immune activation. In contrast, indices of the thymus, heart, and liver did not differ significantly among groups (*p* > 0.05), indicating that PACs exerted no toxic effects on these organs.

Figure 3C shows the dynamic changes in serum-specific IgG antibody levels after booster immunization. On day 7, IgG levels in the low- and high-dose proanthocyanidin groups were significantly higher than those in the Y-CSF group (*p* < 0.05), whereas no significant difference was observed between the medium-dose group and the Y-CSF group (*p* > 0.05). At this time point, the APS group also did not differ significantly from the Y-CSF group (*p* > 0.05), and no significant differences were found between any proanthocyanidin-treated groups and the APS group (*p* > 0.05). As immunization progressed, IgG levels in all groups increased steadily from day 14 to day 35, reaching a peak on day 35. At this stage, IgG levels in all proanthocyanidin dose groups as well as the APS group were significantly higher than those in the Y-CSF group (*p* < 0.05). Notably, IgG levels in the medium- and high-dose proanthocyanidin groups were also significantly higher than those in the APS group (*p* < 0.05), further supporting their synergistic immunoenhancing effects. During the antibody maintenance phase from days 42 to 84 after the booster immunization, overall IgG levels showed a declining trend, but the medium-dose group consistently maintained higher IgG concentrations than the other groups. By day 84, no significant differences were observed between the Y-CSF group and the APS group, or the low- and high-dose proanthocyanidin groups (*p* > 0.05). In contrast, IgG levels in the medium-dose group remained significantly higher than those in the Y-CSF group (*p* < 0.05) and were also significantly elevated compared with the APS group (*p* < 0.05). No significant differences were found between the low- and high-dose groups and the APS group (*p* > 0.05). These findings indicate that PACs enhanced the specific IgG response induced by the CSFV subunit vaccine, with the medium dose showing the most stable and long-lasting immunoenhancing effect.

Figure 3D further evaluated the effects of different treatments on IgG subclass expression. For IgG1, only the medium-dose proanthocyanidin group exhibited a significant increase compared with the Y-CSF group (*p* < 0.05), while the APS group and the low- and high-dose groups showed upward trends without statistical significance (*p* > 0.05). For IgG3, both the medium- and high-dose groups were highly and significantly increased relative to the Y-CSF group (*p* < 0.01), whereas the APS group showed a non-significant upward trend. However, no significant differences were observed between any of the proanthocyanidin groups and the APS group (*p* > 0.05). For IgG2a, the APS group, medium-dose group, and high-dose group were all highly significantly elevated compared with the Y-CSF group (*p* < 0.01), while the low-dose group also showed a significant increase (*p* < 0.05). Compared with the APS group, IgG2a levels in the medium- and high-dose groups were significantly higher (*p* < 0.01). For IgG2b, the APS group and the medium- and high-dose groups were all highly significantly elevated relative to the Y-CSF group (*p* < 0.01), while the low-dose group showed a significant increase (*p* < 0.05). Compared with the APS group, the medium- and high-dose groups were highly significantly increased (*p* < 0.01), and the low-dose group also showed a significant increase (*p* < 0.05); however, no significant difference was found between the high-dose group and the APS group (*p* > 0.05).

Taken together, these results demonstrate that PACs broadly enhanced the expression of IgG1, IgG3, IgG2a, and IgG2b, thereby exhibiting strong potential for humoral immune enhancement. Overall, PACs displayed favorable adjuvant properties when combined with the CSFV subunit vaccine. Without affecting body weight or the safety of major organs, PACs significantly increased the splenic index and promoted the expression of specific IgG and its subclasses. Among them, the medium-dose group showed superior performance compared with the APS group in terms of IgG peak maintenance, breadth of subclass expression, and magnitude of antibody enhancement, highlighting its potential as an adjuvant in CSFV subunit vaccine immunization strategies.

### 2.4. Effects of Pacs on Immune Responses in Mice Immunized with a Subunit Vaccine Against Csfv

**Figure 4:**
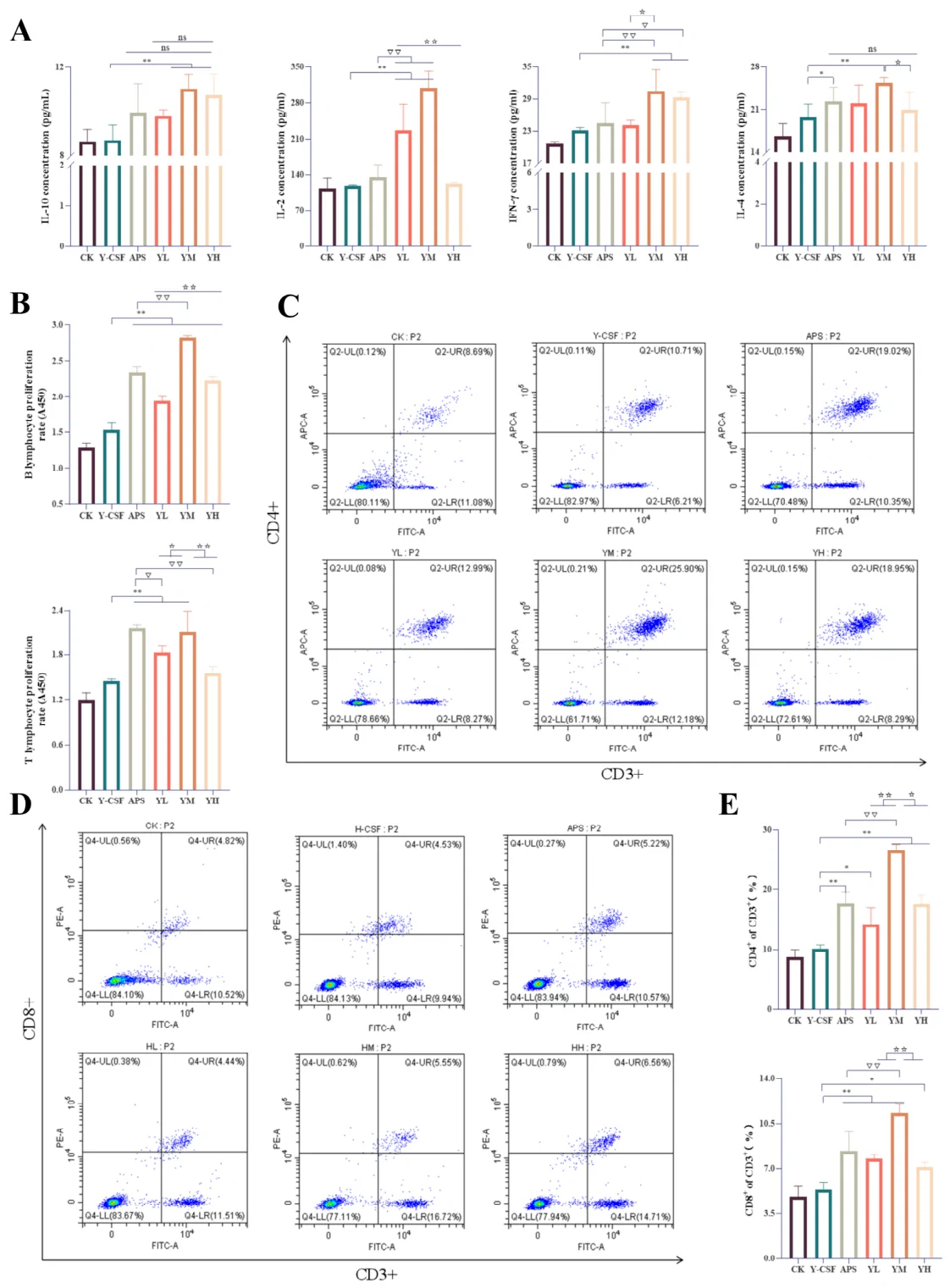
Effects of PACs on immune responses in mice immunized with a subunit vaccine against CSFV. (**A**) Serum concentrations of IL-10, IL-2, IFN-γ, and IL-4 on day 35 after booster immunization. (**B**) Proliferation rates of splenic B lymphocytes and T lymphocytes on day 35 after booster immunization. (**C**,**D**) Flow cytometric analysis of CD4^+^ (**C**) and CD8^+^ (**D**) T-cell subsets in peripheral blood. In the pseudocolor plots, cooler and warmer colors indicate lower and higher event densities, respectively. (**E**) Ratios of CD4^+^/CD3^+^ and CD8^+^/CD3^+^ T cells in peripheral blood on day 35 after booster immunization. Note: compared with the Y-CSF group, * *p* < 0.05, ** *p* < 0.01; compared with the APS group, ^▽^ *p* < 0.05, ^▽▽^ *p* < 0.01; compared with the YM group, ^☆^ *p* < 0.05, ^☆☆^ *p* < 0.01.

ELISA results (Figure 4A) showed that combined immunization with PACs and the CSFV subunit vaccine significantly affected serum cytokine levels in mice. Compared with the Y-CSF group, IL-4 levels were significantly increased in the APS group (*p* < 0.05) and highly significantly increased in the medium-dose proanthocyanidin group (*p* < 0.01). Although all proanthocyanidin groups showed upward trends compared with the APS group, the differences were not statistically significant (*p* > 0.05). For IL-10, both the medium- and high-dose proanthocyanidin groups were highly and significantly higher than the Y-CSF group (*p* < 0.01), while no significant differences were observed in the other groups (*p* > 0.05). All proanthocyanidin groups showed increasing trends relative to the APS group, but without statistical significance (*p* > 0.05). For IL-2, the medium- and high-dose proanthocyanidin groups were highly significantly elevated compared with both the Y-CSF and APS groups (*p* < 0.01), while the other groups showed non-significant upward trends. For IFN-γ, the medium- and high-dose proanthocyanidin groups were highly significantly higher than the Y-CSF group (*p* < 0.01). Moreover, IFN-γ levels in the medium-dose group were also highly significantly higher than those in the APS group (*p* < 0.01), whereas the other groups showed no significant differences (*p* > 0.05). Taken together, these results suggest that PACs enhanced both Th1-type cytokines (IL-2 and IFN-γ) and Th2-type cytokines (IL-4 and IL-10), thereby inducing a mixed Th1/Th2 immune response.

As shown in Figure 4B, splenic lymphocyte proliferation was markedly affected by combined immunization with PACs and the CSFV subunit vaccine. Compared with the Y-CSF group, both the APS group and all proanthocyanidin dose groups significantly promoted B lymphocyte proliferation (*p* < 0.01). Further comparisons revealed that the low- and medium-dose proanthocyanidin groups induced significantly greater B-cell proliferation than the APS group (*p* < 0.01), whereas no significant difference was observed between the high-dose group and the APS group (*p* > 0.05). For T lymphocytes, the APS group and the low- and medium-dose proanthocyanidin groups all showed highly significant increases compared with the Y-CSF group *(p* < 0.01). Compared with the APS group, the low-dose group exhibited a significant increase in T-cell proliferation (*p* < 0.05), while the high-dose group showed a highly significant increase (*p* < 0.01). These findings suggest that different doses of PACs exert differential effects on B- and T-lymphocyte proliferation.

Flow cytometric analysis further revealed changes in T-cell subsets (Figure 4E). For CD3^+^CD4^+^ T cells, the percentages in the APS group and in the medium- and high-dose proanthocyanidin groups were all highly significantly increased compared with the Y-CSF group (*p* < 0.01). The medium-dose group also showed a significant increase relative to the Y-CSF group (*p* < 0.05). Compared with the APS group, the CD4^+^ T-cell proportion in the medium-dose group was highly significantly elevated (*p* < 0.01) and was also significantly higher than those in the low- and high-dose proanthocyanidin groups (*p* < 0.01), demonstrating the superior capacity of the medium dose to activate helper T cells. For CD3^+^CD8^+^ T cells, the APS group and the low- and medium-dose proanthocyanidin groups exhibited highly significant increases compared with the Y-CSF group (*p* < 0.01), while the high-dose group showed a significant increase (*p* < 0.05). Relative to the APS group, the medium-dose group displayed a highly significant increase in the percentage of CD8^+^ T cells (*p* < 0.01), and this was also significantly higher than the low- and high-dose groups (*p* < 0.01). These results indicate that medium-dose PACs exert the strongest effect in promoting cytotoxic T-cell differentiation.

### 2.5. Adjuvant Effects of Pacs on Different Csfv Vaccines

**Figure 5:**
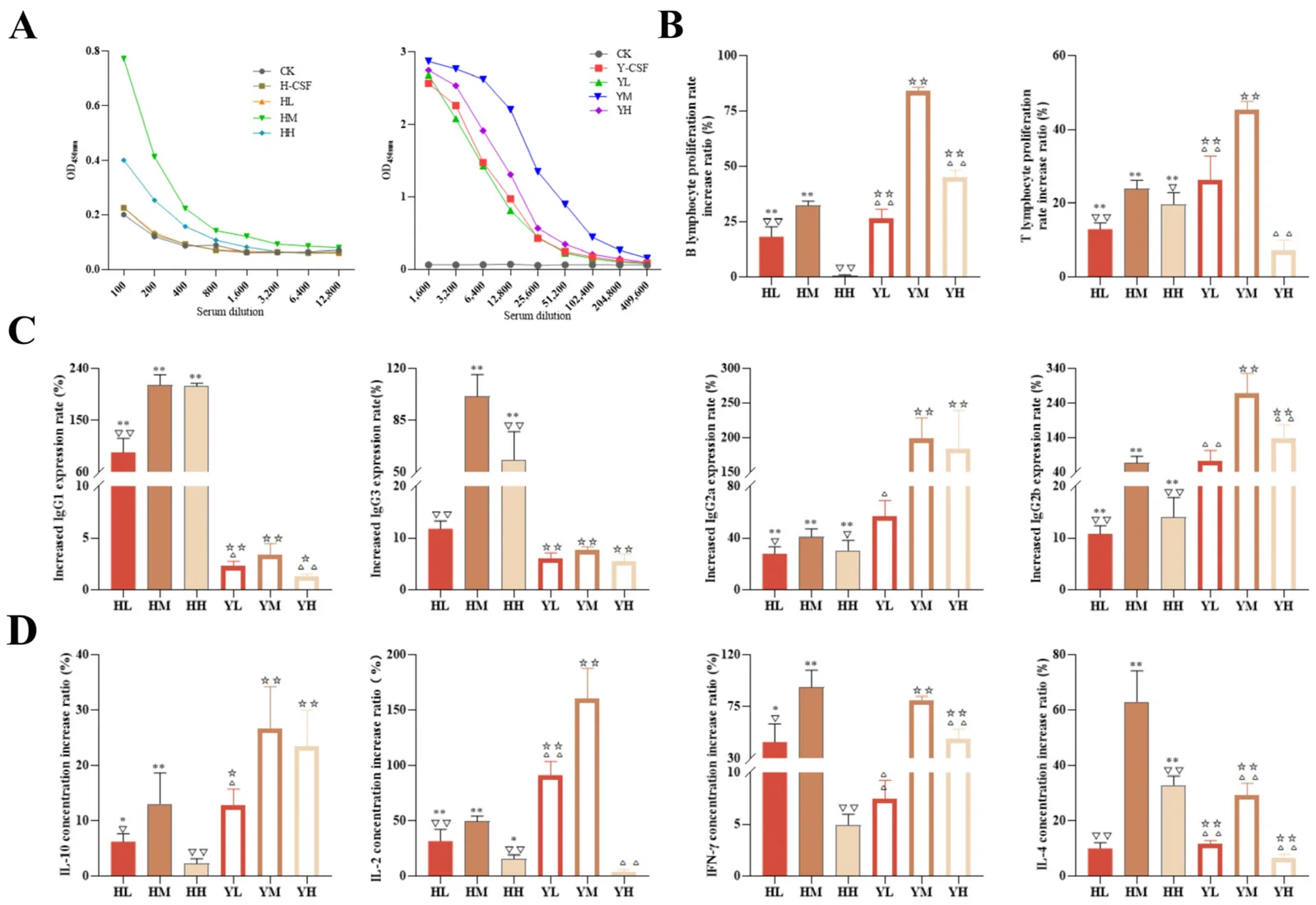
Adjuvant effects of PACs on different CSFV vaccines. (**A**) Serum IgG antibody titers in mice immunized with different CSFV vaccines combined with proanthocyanidin treatment at various time points. (**B**) Proliferation rates of splenic B lymphocytes and T lymphocytes in mice on day 35 after immunization with different CSFV vaccines combined with PACs. (**C**) Relative expression of serum IgG subclasses (IgG1, IgG3, IgG2a, and IgG2b) in mice on day 35 after immunization with different CSFV vaccines combined with PACs. (**D**) Relative serum concentrations of cytokines IL-10, IL-2, IFN-γ, and IL-4 in mice on day 35 after immunization with different CSFV vaccines combined with PACs. Note: Compared with H-CSF group, * *p* < 0.05, ** *p* < 0.01; Compared with HM group, ^▽^ *p* < 0.05, ^▽▽^ *p* < 0.01, compared with Y-CSF group, ^☆^ *p* < 0.05; ^☆☆^ *p* < 0.01; Compared with YM group, ^△^ *p* < 0.05, ^△△^ *p* < 0.01.

To compare the modulatory effects of different doses of PACs on humoral immunity induced by the attenuated and subunit CSFV vaccines, serum antibody titers were first measured (Figure 5A). Under attenuated vaccine immunization, at serum dilutions of 1:100–1:400, antibody titers in the medium- and high-dose proanthocyanidin groups were significantly higher than those in the other groups. At a dilution of 1:800, titers in the vaccine and low-dose groups were even lower than those in the blank control group, and at dilutions ranging from 1:3200 to 1:12,800, titers in all immunized groups were nearly equivalent to those in the blank control group. In contrast, under subunit vaccine immunization, all four immunized groups produced strong specific antibody responses, which were significantly higher than those in the blank control group. Among these, the medium-dose proanthocyanidin group achieved the highest antibody titer (1:409,600), which was significantly greater than those of the vaccine group (1:102,400), the low-dose group (1:102,400), and the high-dose group (1:204,800). No antibodies were detected in the blank group. At serum dilutions of 1:1600–1:102,400, antibody titers in the medium-dose group consistently remained significantly higher than those in the other immunized groups. At higher dilutions of 1:51,200–1:409,600, antibody titers in the vaccine, low-dose, and high-dose groups tended to converge. Taken together, PACs enhanced the immune response induced by the attenuated vaccine by approximately 3.4-fold, whereas the enhancement observed with the subunit vaccine reached approximately 4-fold (endpoint titer 1:409,600 vs. 1:102,400), suggesting that PACs exert a more pronounced immunoenhancing effect in the context of subunit vaccination.

Further analysis of splenic B- and T-lymphocyte proliferation is shown in Figure 5B. For B cells, compared with the attenuated vaccine group, both the low- and medium-dose proanthocyanidin groups significantly promoted B-cell proliferation, with increases of 18.27% and 32.40%, respectively (*p* < 0.01). Under subunit vaccine immunization, all proanthocyanidin dose groups showed highly significant increases in B-cell proliferation, with increases of 26.36%, 83.95%, and 45.25% for the low-, medium-, and high-dose groups, respectively (*p* < 0.01). Notably, the proliferation rate in the medium-dose group was 51.55% higher than that induced by the attenuated vaccine. For T cells, compared with the attenuated vaccine group, the low-, medium-, and high-dose proanthocyanidin groups significantly enhanced proliferation, with increases of 12.97%, 23.99%, and 19.80%, respectively (*p* < 0.01). Relative to the subunit vaccine group, both the low- and medium-dose groups also showed significant increases of 26.26% and 45.46%, respectively (*p* < 0.01). Moreover, the proliferation rate in the medium-dose group was significantly higher than that in the low-dose group (*p* < 0.01), indicating that the medium dose was the most effective in promoting cellular immune responses.

Analysis of antibody subclasses (Figure 5C) showed that, compared with the attenuated vaccine group, all three proanthocyanidin dose groups significantly increased IgG1 levels by 94.46%, 211.49%, and 209.31%, respectively (*p* < 0.01). Relative to the subunit vaccine group, the low- and medium-dose groups showed highly significant increases of 2.34% and 3.43%, respectively (*p* < 0.01), while the high-dose group showed a significant increase (*p* < 0.05). Notably, the IgG1 level in the medium-dose group combined with the attenuated vaccine was significantly higher than that combined with the subunit vaccine, with an increase of 208.06% (*p* < 0.01). For IgG2a, all three proanthocyanidin dose groups exhibited significant increases of 27.94%, 41.08%, and 30.30%, respectively (*p* < 0.05). The most pronounced increases were observed when medium and high doses were combined with the subunit vaccine, with levels elevated by 199.40% and 184.36%, respectively (*p* < 0.01). For IgG2b, all proanthocyanidin dose groups showed significant increases (*p* < 0.01), with the most striking enhancements observed in the medium- and high-dose groups combined with the subunit vaccine (269.09% and 138.17%, respectively; *p* < 0.01). For IgG3, the medium- and high-dose groups combined with the attenuated vaccine exhibited highly significant increases of 101.14% and 58.51%, respectively (*p* < 0.01), and the medium-dose group was significantly higher than both the low- and high-dose groups (*p* < 0.01). Compared with the subunit vaccine group, all three proanthocyanidin dose groups showed significant increases in IgG3 (*p* < 0.01), though the magnitude of elevation was relatively smaller. Overall, PACs exerted the most pronounced enhancing effects on IgG2a and IgG2b when combined with the subunit vaccine, while their effect on IgG3 enhancement was stronger when combined with the attenuated vaccine.

**Figure 6:**
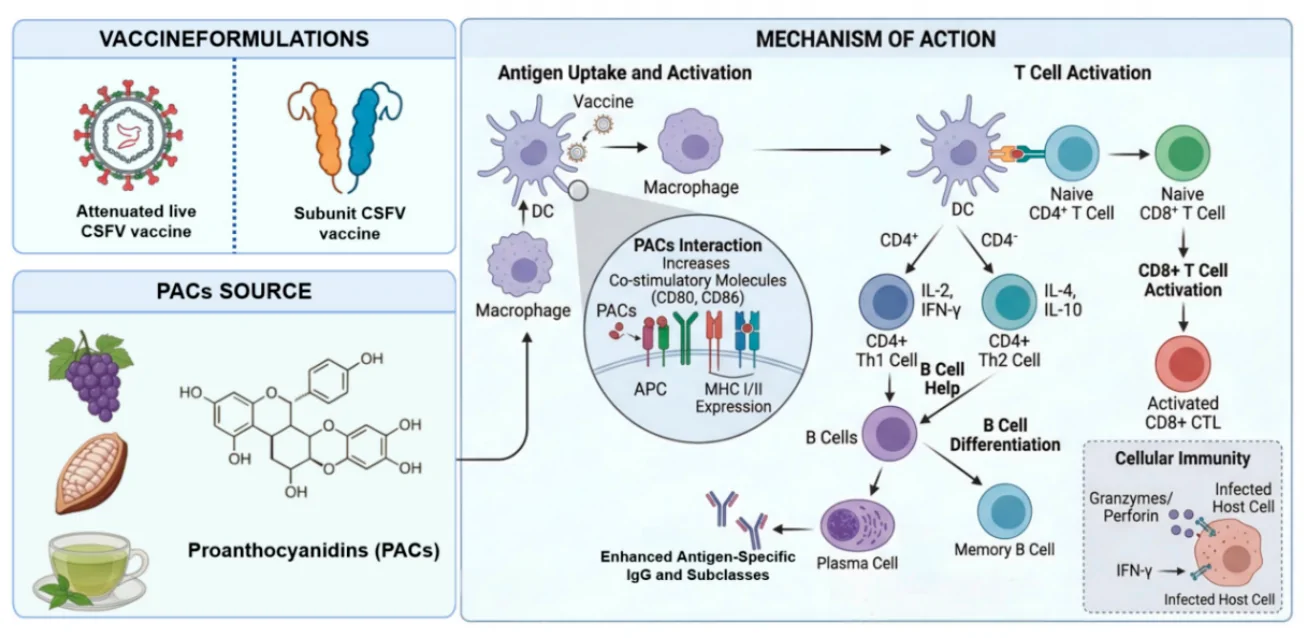
Proanthocyanidins as natural vaccine adjuvants: sources, formulations, and immunomodulatory mechanisms. Natural proanthocyanidins (PACs) derived from grapes, cocoa, and tea serve as adjuvants for both attenuated live and subunit CSFV vaccines. PACs enhance antigen uptake and activation by dendritic cells and macrophages through increased co-stimulatory molecules (CD80, CD86) and MHC class I/II expression. This promotes balanced Th1 (IL-2, IFN-γ) and Th2 (IL-4, IL-10) responses, leading to enhanced B cell differentiation into plasma cells and memory B cells that produce elevated antigen-specific IgG and subclasses. Concurrently, activated CD8+ T cells provide cellular immunity through granzyme/perforin-mediated cytotoxicity and IFN-γ production against infected cells. Arrows indicate the proposed direction of cellular interactions, differentiation, and immune-response progression. Colors are used solely to distinguish different cell types and functional components and do not represent quantitative differences. The integrated humoral and cellular immune responses demonstrate PACs’ potential as safe and effective natural vaccine adjuvants.

Cytokine comparison results (Figure 5D) showed that, compared with the attenuated vaccine group, medium-dose PACs significantly increased IL-10 levels by 13.04% (*p* < 0.05), whereas the low- and high-dose groups showed no significant differences. Relative to the subunit vaccine group, the medium-dose group also showed a significant increase in IL-10 levels by 26.77% (*p* < 0.05). For IL-4, both the medium- and high-dose groups showed significant increases compared with the attenuated vaccine group (63.05% and 32.86%, respectively; *p* < 0.01). Moreover, IL-4 levels in the medium-dose group were significantly higher than those in the low- and high-dose groups (48.25% and 22.72%, respectively; *p* < 0.01). Compared with the subunit vaccine group, the medium-dose group also exhibited a significant increase in IL-4 by 29.23% (*p* < 0.05). For IL-2, all three proanthocyanidin dose groups showed highly significant increases relative to the attenuated vaccine group (31.78%, 49.19%, and 28.80% for the low-, medium-, and high-dose groups, respectively; *p* < 0.01). The medium-dose group was also highly and significantly higher than the low- and high-dose groups (*p* < 0.01). Compared with the subunit vaccine group, IL-2 levels in the low- and medium-dose groups were significantly increased by 91.08% and 160.60%, respectively (*p* < 0.01). For IFN-γ, the medium-dose group was highly significantly elevated compared with the attenuated vaccine group (91.94% increase; *p* < 0.01) and was also higher than the high-dose group (*p* < 0.01). Compared with the subunit vaccine group, both the medium- and high-dose groups exhibited highly significant increases (79.59% and 47.08%, respectively; *p* < 0.01), with IFN-γ levels in the medium-dose group being significantly higher than those in the low- and high-dose groups (*p* < 0.01). Overall, the medium-dose group demonstrated the most pronounced effect in promoting IL-2 and IFN-γ secretion, suggesting a strong activating effect on Th1-type immunity. The principal immunological outcomes observed in this study are summarized schematically in Figure 6.

## 3. Discussion

Against the dual backdrop of increasingly stringent global requirements for vaccine safety and growing pressure regarding public acceptance of vaccines, exploring safe strategies that holistically optimize immune response capabilities at the host level has become a key focus in translational vaccine research. Unlike injectable adjuvants incorporated into vaccine formulations, oral immunomodulators may offer a convenient complementary approach for influencing vaccine-associated immune responses. As natural, food-derived oral immunomodulators, PACs possess core advantages such as inherent safety, easy accessibility, excellent biocompatibility, and multi-target immunomodulatory activity [16,17], thereby meeting the fundamental requirements of immunostimulation strategies. In this study, we utilized a CSFV model to systematically evaluate the feasibility and efficacy of oral PACs in enhancing vaccine-induced immune responses—this virus belongs to the Flaviviridae family, and its immunological characteristics are highly similar to those of clinically significant human pathogens such as hepatitis C virus and dengue virus. The results showed that oral PACs at 30 mg/kg/day, particularly when combined with the subunit vaccine, were associated with the most consistent increases in the measured antibody, cytokine, lymphocyte-proliferation, and T-cell-subset outcomes, without detectable changes in body weight or the examined organ indices. These findings provide solid empirical support for the development of naturally active ingredients derived from food sources as oral immunostimulants and highlight the core value of the strategy of combining oral PACs with vaccination—a key advantage that was further validated in subsequent safety and immunogenicity analyses. The relevance of these CSFV findings to human hepatitis C virus or dengue virus nevertheless remains a hypothesis that requires direct validation.

Safety is a core prerequisite for the widespread adoption of oral immunostimulation strategies and represents their primary advantage over traditional injectable adjuvants. Subunit vaccines, characterized by high purity, low risk of inactivation, and broad cross-species applicability, have become the mainstream approach in vaccine development against hepatitis B virus (HBV), human papillomavirus (HPV), coronaviruses, and other pathogens [18]. However, subunit vaccines exhibit relatively weak immunogenicity; while traditional adjuvants can compensate for this shortcoming, their safety concerns directly impact the feasibility of mass vaccination and public acceptance. This study demonstrates that oral PACs, within a dosage range of 15–60 mg/kg/day, whether administered alone or in combination with attenuated live vaccines or subunit vaccines, did not cause any organic damage to the body weight gain or vital organs (heart, liver, thymus) of the test mice, and their safety window fully covers the effective dosage range. In contrast, traditional injectable adjuvants have multiple limitations: aluminum adjuvants often induce local granulomas, chronic inflammatory accumulation, and potential neurotoxicity [19,20,21]; emulsion-based adjuvants frequently cause redness, induration, and persistent tissue damage at the injection site; while Toll-like receptor agonists are prone to triggering systemic cytokine storms and fever-related adverse reactions [22]. The oral route of administration fundamentally avoids injection-related local adverse reactions, a characteristic that is particularly important for vaccine applications requiring mass vaccination. The spleen is a major secondary lymphoid organ, and the spleen index is commonly used as a general indicator of immune-organ status in mice. In the present study, the increased spleen index in several PAC-treated groups was accompanied by enhanced splenic B- and T-lymphocyte proliferation, elevated antibody and cytokine responses, and changes in peripheral CD4^+^ and CD8^+^ T-cell proportions. Similar findings have been reported for natural immunomodulators, including polysaccharides and proanthocyanidins, in which increased spleen indices were associated with enhanced splenocyte proliferation, immunoglobulin production, cytokine secretion, or T-cell responses [23,24,25]. These concordant observations suggest that the increased spleen index may reflect enhanced splenic immune responsiveness. This indicates that orally absorbed PACs can systemically reach immune organs, activating the proliferation and differentiation of innate immune cells in a gentle, low-damage manner, thereby regulating the host immune system to an optimal state for responding to vaccine antigens. This targeted regulation of immune organs via the oral route ensures the precision of immune enhancement while maintaining good safety and compliance, laying the foundation for subsequent vaccine-induced immune enhancement effects. It should be noted, however, that the increased splenic index was not corroborated by histopathology and therefore cannot by itself distinguish immune activation from pathological enlargement.

PAC administration produced a larger relative increase in several measured immune readouts in the subunit vaccine model than in the live-attenuated vaccine model, particularly for antigen-specific IgG. Subunit vaccines offer high safety [26], but their core limitation lies in containing only a single purified antigen, lacking viral replication capacity and the innate immune activation signals provided by pathogen-associated molecular patterns (PAMPs). When used alone, they often induce only weak antibody responses and transient immune protection [27,28]. Therefore, enhancing the host’s immune response to subunit vaccines through exogenous means is a key strategy to overcome this bottleneck. Our data clearly demonstrate that when moderate doses of oral PACs are administered in conjunction with subunit vaccine vaccination, peak titers of specific IgG antibodies in the serum of test animals can reach 1:409,600, representing a fourfold increase compared to the group receiving subunit vaccine alone. This enhancement effect is significantly higher than that observed in the group receiving oral PACs in combination with live attenuated vaccines (3.4-fold), and there are significant statistical differences among the groups. The immunological basis for this difference lies in the fact that attenuated live vaccines retain viral replication capacity, enabling sustained antigen expression and activation of multiple pattern recognition receptors, thereby possessing inherent strong immunogenicity [29,30]; consequently, the scope for enhancing the immunomodulatory effects of oral PACs is relatively limited. The greater relative enhancement observed in the subunit vaccine model may partly reflect its lower baseline immunogenicity and the correspondingly greater scope for improvement. However, the biological basis of this difference remains to be determined. Because the underlying mechanism—including intestinal absorption and microbial metabolism of PACs and direct dendritic-cell activation—was not directly examined, “host immune conditioning” is used here as a descriptive rather than a mechanistically demonstrated concept, and the present design cannot fully distinguish antigen-specific enhancement from non-specific immunostimulation.

The concurrent increases in Th1-associated cytokines (IL-2 and IFN-γ) and Th2-associated cytokines (IL-4 and IL-10), together with changes in IgG subclasses, are consistent with a mixed Th1/Th2-associated immune profile. However, these serum cytokine and antibody measurements do not directly demonstrate antigen-specific Th1/Th2 differentiation or protective antiviral immunity. When traditional commercial adjuvants (such as aluminum adjuvants) are used in combination with subunit vaccines, they unidirectionally activate the Th2 humoral immune pathway, relying entirely on neutralizing antibodies to control free viral particles, while failing to activate intracellular viral clearance pathways [31,32]. Consequently, they cannot meet the requirements for controlling severe viral infections. Specific IgG subclass analysis in this study confirmed that oral PACs administered in conjunction with subunit vaccination not only effectively upregulate protective antibody levels of the Th2 pathway’s downstream subclasses IgG1 and IgG3—thereby enhancing humoral neutralizing capacity—but also achieve a breakthrough increase in the expression of the Th1 pathway’s core subclasses IgG2a and IgG2b, with increases of 199.40% and 269.09%, respectively, thereby precisely compensating for the deficiencies of subunit vaccines in cellular immunity. It should be noted that the murine IgG subclass framework linking IgG2a/IgG2b to Th1 and IgG1 to Th2 is strain-dependent (the IgG2a/IgG2c allotype of Kunming mice should be taken into account); the elevated IgG2a/IgG2b are therefore interpreted here as indirect indicators rather than direct proof of enhanced cellular immunity. Functionally, IgG1 effectively blocks viral invasion of susceptible cells, while IgG2a activates the complement cascade and antibody-dependent cellular cytotoxicity (ADCC), thereby targeting and the lysis of virus-infected cells, achieving synergistic protection between humoral and cellular immunity [33,34,35]. Furthermore, 84 days post-vaccination, specific IgG levels in the group receiving oral PACs in combination with vaccination remained significantly higher than those in the vaccine-only group and other control groups, indicating that oral PACs can promote long-term immune protection following vaccination [36]. These findings further highlight the unique advantages of oral PACs as an immunomodulatory strategy when used in conjunction with vaccination. It should also be noted that no head-to-head comparison with a conventional adjuvant (e.g., aluminium salts) was performed, and that only young female Kunming mice were studied, which limits generalizability across sex, age, species, and adjuvant class.

The coordinated changes in serum cytokines, IgG subclasses, splenic lymphocyte proliferation, and peripheral T-cell proportions provide a descriptive immunological profile of the responses associated with oral PAC administration. However, the present study did not identify the molecular pathways responsible for these changes. Quantitative cytokine analysis indicates that oral PACs precisely upregulate the beneficial expression of IL-4 and IL-10: IL-4, a key factor in Th2 differentiation, promotes B-cell proliferation and antibody class switching [3,37], which is highly consistent with the observed increase in IgG1 levels; IL-10 promotes B-cell survival while suppressing excessive inflammatory responses, thereby maintaining immune homeostasis and preventing immunopathological damage, which aligns with the mild, low-toxicity safety profile of oral PACs [38]. Concurrently, oral PACs significantly increased the expression levels of IFN-γ and IL-2: IFN-γ activates macrophages, upregulates MHC molecule expression, and induces IgG2a class switching in B cells [39,40]; the 91.94% increase in IFN-γ is consistent with, and may contribute to, the significant elevation of IgG2a. As a T-cell growth factor, IL-2 drives T-cell clonal expansion and the generation of memory T cells [41]; the 160.60% increase parallels the enhanced T-cell proliferation. Flow cytometry analysis revealed that oral PACs combined with subunit vaccination simultaneously increased the proportions of both CD4^+^ helper T cells and CD8^+^ cytotoxic T cells, thereby synergistically enhancing humoral immunity and cytotoxic effects [42,43]. This differs from the pattern observed with oral PACs combined with live-attenuated vaccines, which primarily activate CD8^+^ T cells. This difference may reflect vaccine-specific immune-enhancing mechanisms: attenuated live vaccines directly activate the CD8^+^ T cell pathway through the virus’s inherent replicative capacity, whereas subunit vaccines primarily rely on cytokines produced by CD4^+^ T cells to indirectly induce CD8^+^ T cell responses [44,45,46]. Therefore, the results suggest that oral PAC administration may be particularly useful for increasing selected immune readouts elicited by a relatively low-immunogenicity subunit vaccine. Notably, this dose–response was non-monotonic: the medium dose (30 mg/kg/d) was optimal for most humoral and Th2-associated readouts, whereas the high dose more strongly favored CD8^+^ T-cell responses—a bell-shaped pattern commonly observed for dietary polyphenols—so a single “optimal dose” should not be over-generalized across all immune endpoints.

## 4. Materials and Methods

### 4.1. Animal Experiment

Animals and Experimental Design:

Specific-pathogen-free (SPF) female Kunming mice (4–6 weeks old, 20–28 g) were purchased from the Henan Provincial Laboratory Animal Center. After a 7-day acclimation period, mice were used for experiments. Housing conditions were maintained under a 12 h light/dark cycle, temperature of 25 ± 3 °C, and relative humidity of 50–60%, with free access to food and water and adequate ventilation. No apparent adverse reactions were observed during acclimation. All animal experimental procedures were reviewed and approved by the Animal Welfare and Ethics Research Committee of the College of Veterinary Medicine, Henan Agricultural University (protocol No. HMND2024110701; approval date: 30 October 2024). The proanthocyanidins (PACs; procyanidins; CAS No. 4852-22-6; Cat. No. SP8520) were purchased from Beijing Solarbio Science & Technology Co., Ltd. (Beijing, China). According to the manufacturer’s specification, the PAC preparation was supplied as a UV-grade mixture with a stated purity of ≥95% as determined by UV assay. The attenuated inactivated vaccine used in this study was a commercially available classical swine fever live vaccine (tissue culture derived) manufactured by Wuhan Keqian Biotechnology Co., Ltd. (Wuhan, China). This vaccine contains a rabbit-attenuated classical swine fever virus strain, CVCC AV1412. The subunit vaccine was a commercially available classical swine fever virus E2 protein recombinant baculovirus inactivated vaccine (WH-09 strain) manufactured by the same company.

Attenuated CSFV vaccine experiment:

A total of 66 mice were randomly assigned using a random number table (*n* = 11 per group) into the following groups: blank control (CK), vaccine control (H-CSF), positive control (vaccine + Astragalus polysaccharide, APS), and three vaccine + proanthocyanidin groups with high (HH, 60 mg/kg/d), medium (HM, 30 mg/kg/d), and low (HL, 15 mg/kg/d) doses. PACs and APS were administered orally by gavage at 0.1 mL/10 g body weight once daily for 4 consecutive days. Mice in the CK and H-CSF groups received an equal volume of saline. Twenty-four hours after the final gavage, each mouse was immunized subcutaneously with a commercial classical swine fever vaccine, live (tissue culture origin; Wuhan Keqian Biology Co., Ltd., Wuhan, China). The vaccine contained the lapinized attenuated CSFV strain CVCC AV1412, with no fewer than 750 rabbit infectious doses per porcine dose. Immediately before use, the lyophilized vaccine was reconstituted with sterile physiological saline according to the manufacturer’s instructions to a final concentration of one porcine dose per milliliter. Each mouse received 0.2 mL of the reconstituted vaccine, corresponding to 0.2 porcine doses and no fewer than 150 rabbit infectious doses. A booster immunization was administered 14 days after the primary immunization using the same vaccine preparation, dose, injection route, and anatomical site.

CSFV subunit vaccine experiment:

Another 66 mice were randomly assigned using a random number table (*n* = 11 per group) to the following groups: blank control (CK), vaccine control (Y-CSF), positive control (vaccine + Astragalus polysaccharide, APS), and three vaccine + proanthocyanidin groups with high (YH, 60 mg/kg/d), medium (YM, 30 mg/kg/d), and low (YL, 15 mg/kg/d) doses. The administration method was the same as that used in the attenuated vaccine experiment. Twenty-four hours after the final gavage, each mouse was immunized subcutaneously with a commercial classical swine fever virus E2 protein recombinant baculovirus vaccine, inactivated (strain WH-09; Wuhan Keqian Biology Co., Ltd., Wuhan, China). The vaccine contained recombinant CSFV E2 protein expressed using a baculovirus expression system. According to the manufacturer’s specification, each 2 mL porcine dose contained 50 μg of recombinant E2 protein, corresponding to an E2 protein concentration of 25 μg/mL. Before administration, the vaccine was equilibrated to room temperature and thoroughly mixed to obtain a uniform suspension. Each mouse received 0.2 mL of the vaccine, corresponding to approximately 5 μg of recombinant E2 protein. A booster immunization was administered 14 days after the primary immunization using the same vaccine preparation, dose, and administration route.

Animal allocation and experimental timeline:

For each vaccine experiment, the 11 mice in each treatment group were prospectively assigned to three sampling subsets before the first intervention. The longitudinal serology subset consisted of three ear-tagged mice per group and was used for repeated survival blood collection at 7-day intervals after booster immunization. These mice provided the serial serum samples used to evaluate the temporal antigen-specific IgG response; serum obtained on day 35 was also used for antibody-titer and IgG-subclass analyses.

A second subset of three mice per group was assigned to the day-35 immunological assessment. On day 35 after booster immunization, peripheral blood was collected from these mice for serum cytokine measurements and flow-cytometric analysis of CD3^+^CD4^+^ and CD3^+^CD8^+^ T-cell proportions. The mice were subsequently euthanized on the same day, and their spleens were aseptically collected for mitogen-induced B- and T-lymphocyte proliferation assays.

The remaining five mice per group constituted the day-84 physiological assessment subset. Their body weights were monitored throughout the experiment, and they were euthanized on day 84 after booster immunization for collection and weighing of the heart, liver, kidney, spleen, and thymus. The longitudinal serology mice were also euthanized after the final blood collection on day 84. Thus, three mice per group were euthanized on day 35, and the remaining eight mice per group were euthanized on day 84. No animals were euthanized at other time points. No unexpected deaths or exclusions occurred during the experiment.

### 4.2. Sample Collection

From the first immunization onwards, body weight of mice was recorded weekly until day 84 after the booster immunization to monitor weight changes and calculate relative body weight percentages. On day 84 after the booster immunization, mice in each group were euthanized and dissected. The heart, liver, kidney, spleen, and thymus were collected, weighed, and used to calculate organ coefficients. During the immunization period, blood samples were collected from the tail vein every 7 days. After clotting at room temperature, samples were centrifuged at 3000 rpm for 10 min at 4 °C, and the serum was separated and stored at −20 °C for subsequent analyses of antibody levels, antibody subclasses, and cytokines. Splenic tissues were processed under aseptic conditions to prepare single-cell suspensions, followed by red blood cell lysis, and used for lymphocyte proliferation assays and related immunological analyses. Peripheral blood samples were used for flow cytometric analysis to determine the proportions of T lymphocyte subsets, including CD4^+^ and CD8^+^ T cells.

### 4.3. Detection of Specific Antibodies and Subclasses

The detection of specific antibodies and their subclasses was performed with reference to the method of Li et al. [47], with appropriate modifications. Sera were collected every 7 days from day 7 to day 84 after the booster immunization (days 7, 14, 21, 28, 35, 42, 49, 56, 63, 70, 77, and 84). At each time point, the same three ear-tagged representative mice per group were sampled from the tail vein (survival bleeding), consistent with the repeated-measures design. Serum was separated and used to measure specific antibody levels and subclasses. Purified CSFV E2 protein (Wuhan Keqian, Wuhan, China, 1 μg/mL) was used as the coating antigen. Each well of a 96-well plate was coated overnight at 4 °C with 100 μL of purified CSFV E2 protein at 1 μg/mL, corresponding to 0.1 μg of E2 protein per well. After washing, 100 μL of blocking buffer (PBST, Solarbio, Beijing, China) was added to each well and incubated at 37 °C for 2 h, followed by washing. Mouse sera were diluted 1:25,600 in PBS (Gibco RPMI-1640, Grand Island, NY, USA) and added to the wells (100 μL per well), then incubated at 37 °C for 30 min. Subsequently, horseradish peroxidase (HRP)-conjugated anti-mouse IgG (Jackson ImmunoResearch, West Grove, PA, USA, 1:3000) or subclass-specific secondary antibodies against IgG1, IgG3, IgG2a, and IgG2b (Jackson ImmunoResearch) were added, followed by incubation at 37 °C for 30 min. After washing, 3,3′,5,5′-tetramethylbenzidine (TMB; Bioss, Woburn, MA, USA) substrate solution was added and incubated in the dark at 37 °C for 10 min. The reaction was stopped with 2 M H_2_SO_4_, and absorbance was measured at 450 nm (OD_450_).

### 4.4. Detection of Cytokines in Mice

On day 35 after the booster immunization, blood samples (~0.5 mL per mouse) were collected from the tail vein of three randomly selected mice in each group. Samples were kept at room temperature for 1 h, stored at 4 °C for 12 h, and then centrifuged at 2000 rpm for 15 min to separate serum. Serum samples were equilibrated to room temperature for approximately 20 min before testing. Serum cytokine levels (IL-4, IL-10, IL-2, and IFN-γ) were measured using commercial ELISA kits (Xinbosheng, Shenzhen, China). Briefly, standards or diluted samples were added to wells of 96-well plates and incubated at 37 °C for 90 min in the dark. After five washes, 100 μL of biotinylated antibody working solution was added to each well and incubated at 37 °C for 60 min in the dark. Plates were washed five times again, followed by the addition of 100 μL of enzyme-conjugate working solution per well and incubation at 37 °C for 30 min. After repeated washing, 100 μL of TMB substrate was added to each well and incubated at 37 °C for 15 min in the dark. The reaction was stopped with 100 μL of 2 M H_2_SO_4_. Absorbance was measured at 450 nm (OD_450_), and cytokine concentrations were calculated from standard curves using a quadratic polynomial equation.

### 4.5. Detection of Splenic T Lymphocyte Proliferation

After the booster immunization, mice were euthanized, and spleens were aseptically collected following disinfection by immersion in 75% ethanol. Spleens were ground and filtered through a 200-mesh copper mesh to obtain single-cell suspensions, which were centrifuged to remove debris. Lymphocytes were isolated using a lymphocyte separation medium (Solarbio, Beijing, China) by density gradient centrifugation at 900× *g* for 30 min. The mononuclear cell layer was collected, washed three times with PBS, and resuspended in complete RPMI-1640 medium (Gibco Life Technologies) supplemented with 10% fetal bovine serum (FBS) to prepare a splenic lymphocyte suspension at a concentration of 2.5 × 10^6^ cells/mL.

Cell suspensions were seeded into 96-well culture plates at 100 μL per well. Blank wells received RPMI-1640 medium alone, while other wells were stimulated with either concanavalin A (ConA, 5 μg/mL) or lipopolysaccharide (LPS, 5 μg/mL). Each treatment was performed in triplicate. Cells were incubated at 37 °C in a 5% CO_2_ incubator for 24 h. One hour before the end of incubation, 10 μL of CCK-8 reagent (Bioss) was added to each well and further incubated. Absorbance at 450 nm (OD_450_) was then measured. The lymphocyte proliferation index (SI) was calculated using the following formula: SI = (ODexperimental − ODblank)/(ODcontrol − ODblank) × 100%.

### 4.6. Detection of T Lymphocyte Subsets

Peripheral blood was collected from mice after the booster immunization and treated with EDTA as an anticoagulant. For each sample, 100 μL of anticoagulated blood was transferred into a flow tube, followed by the addition of 5 μL each of FITC-conjugated anti-CD3, APC-conjugated anti-CD4, and PE-conjugated anti-CD8 antibodies (Lianke Bio, Hangzhou, China). Samples were incubated for 15 min at room temperature in the dark. Subsequently, 6–10 volumes of red blood cell lysis buffer (Bioss) were added, and samples were incubated at room temperature for 10 min to lyse erythrocytes. After centrifugation at 300× *g* for 5–8 min, the supernatant was discarded, and the cell pellet was resuspended in 500 μL of PBS. Samples were analyzed using a CytoFlex flow cytometer (Beckman Coulter, Brea, CA, USA), and data were acquired and processed with CytExpert software (version 2.4; Beckman Coulter). The proportions of CD3^+^, CD3^+^CD4^+^, and CD3^+^CD8^+^ T-cell subsets in peripheral blood were determined.

### 4.7. Statistical Analysis

All experimental data were expressed as mean ± standard deviation (mean ± SD). Statistical analyses were performed using SPSS software (version 26.0; IBM, Armonk, NY, USA). Prior to parametric analysis, normality was assessed by the Shapiro–Wilk test and homogeneity of variance by Levene’s test. Comparisons between two groups were conducted using independent samples *t* tests. For comparisons among multiple groups, one-way analysis of variance (ANOVA) was used, followed by Duncan’s or Tukey’s post hoc test for multiple comparisons. Repeated measures ANOVA was applied for data involving different time points. Differences were considered statistically significant at *p* < 0.05 and highly significant at *p* < 0.01. The number of biological replicates (n) per group and the meaning of error bars (SD) are stated in the corresponding figure legends. Graphs were generated using GraphPad Prism software (version 10.6.0; GraphPad Software, Boston, MA, USA).

## 5. Conclusions

Using a murine CSFV vaccination model, this study evaluated the immunological outcomes associated with oral administration of food-derived PACs in combination with live-attenuated or subunit CSFV vaccines. These findings provide crucial experimental evidence and theoretical support for the development of safe and effective oral immune-enhancing strategies. Our findings confirm that oral administration of PACs significantly activates splenic immune function by systemically modulating the host’s immune state, comprehensively enhancing vaccine-induced humoral and cellular immune responses without affecting growth and development or causing organ damage. This is manifested by a significant increase in the levels of specific IgG and its subclasses, increased secretion of key cytokines including IL-4, IL-10, IL-2, and IFN-γ, and a significant increase in the proportions of CD4^+^ and CD8^+^ T cells, collectively achieving a broad-spectrum enhancement of the vaccine-induced immune response. Dose–response analysis indicates that moderate doses of orally administered PACs exhibit the strongest immunostimulatory effect. Notably, in the subunit vaccine model, oral PACs effectively compensated for the insufficient immune response induced by weakly immunogenic vaccines, further highlighting the unique value of oral PACs as an immune-enhancing strategy when used in conjunction with vaccination. In summary, dietary proanthocyanidins (PACs) combine safety, efficacy, and edibility, demonstrating significant potential for use as oral immunostimulants in conjunction with vaccines. This study lays the foundation for the translational application of oral PACs as immunomodulators to enhance vaccine-induced immunity, highlighting the value of natural bioactive food components in optimizing vaccine immunogenicity.

## Data Availability

The original contributions presented in the study are included in the article; further inquiries can be directed to the corresponding authors.
